# The Outcome of Manual Small Incision Cataract Surgery and Anterior Vitrectomy for Persistent Fetal Vasculature in an 18-Year-Old Woman: A One-Year Follow-Up

**DOI:** 10.7759/cureus.10605

**Published:** 2020-09-23

**Authors:** Ejike Egbu

**Affiliations:** 1 Ophthalmology, Lily Hospitals Limited, Warri, NGA

**Keywords:** persistent fetal vasculature, amblyopia, intraocular lens, hyaloid artery, bergmeister's papillae, leukocoria, mittendorf dot, vitrectomy, visual rehabilitation, manual small incision cataract surgery

## Abstract

The surgical management of persistent fetal vasculature (PFV) is challenging and the visual outcome can be compromised by coexisting ocular pathologies and amblyopia. It can be considered for relief of retinal traction and improved cosmetic appeal when a squint or a posterior capsular cataract is present. In the case presented in this report, the intermittent exotropia improved from 45 to 30 degrees in one year, which suggests an improvement in binocular single vision. There was also a resolution of the leukocoria and retinal traction. The patient underwent the following examinations: visual activity, slit-lamp biomicroscopy, intraocular pressure (Goldmann), fundus photography (OCT TOPCON, 3D OCT-1 Maestro, Topcon, Tokyo, Japan), B-scan Doppler ultrasonography (Mindray DC-N3, Mindray, Shenzhen, China), Keratometry (Topcon KR 800, Topcon, Tokyo, Japan), and axial length (Sonomed 300AP+A Scan/Pachymeter, Sonomed Escalon, Lake Success, NY). Intraocular lens (IOL) power was calculated with the Sanders-Retzlaff-Kraff (SRK) II formula. During surgery, a rigid polymethyl methacrylate (PMMA) IOL was inserted into the sulcus after excision of the lesion and anterior vitrectomy. The first day's postoperative evaluation included visual acuity, corneal transparency, depth of anterior chamber, pupil size, shape, pupillary reaction to light, and position of the IOL. Intraocular pressure was normal within the follow-up period. Fundus photography and B-scan examinations were performed at one month and one year.

## Introduction

The fetal vasculature is formed by the hyaloid artery, and supplies the developing lens in intrauterine life [1]. It is most developed around the ninth week of gestation but slowly regresses by the seventh month, leaving a clear central part known as the Cloquet's canal [2]. When the hyaloid vasculature does not regress completely, it leads to the formation of a vestigial structure known as persistent fetal vasculature (PFV), which was previously known as persistent hyperplastic primary vitreous (PHPV) [3,4]. The pathogenesis of PFV is not fully understood, but it is thought to involve one or all of the following mechanisms: lenticular development, genetic factors in familial cases, defects in genes and growth factors that regulate the regression of vessels, and an imbalance between vaso-inhibitory and vaso-stimulatory factors [5]. Two genes, ATOH7 and NDP, have been implicated in autosomal recessive and dominant patterns of inheritance, respectively. Morphologically, PFV can be anterior, posterior, or a combination of both [6]. Anterior PFV is characterized by one, some, or all of the following features: a retrolental opacity (Mittendorf dot), elongated ciliary processes, cataract, shallow anterior chamber, poor pupil dilation, microphthalmos, microcornea, and glaucoma [7] while the posterior PFV has the following features: the stalk of tissue attached to the optic nerve (Bergmeister's papillae), retinal folds, dysplasia, retinal detachment, and varying degrees of optic nerve dysplasia [8,9]. The prevalence rate of PFV is unknown, however, published articles suggest that it is responsible for about 5% of all cases of blindness [10].

Comparing the surgical outcomes of PFV is challenging due to the varying nature of its presentation and depends on the morphological type, associated with ocular pathology, time of diagnosis and surgery, and postoperative amblyopia therapy [8,11]. Surgical interventions should address lenticular opacity, retinal dysplasia and traction, and glaucoma when present in order to achieve a good outcome. Operative techniques for PFV include the following: anterior limbal lensectomy, pars plana lensectomy, membranectomy, vitrectomy, and trabeculotomy [3,12]. Even after a successful surgical operation, the visual function may still be suboptimal, so continued follow-up is essential. Visual rehabilitation postoperatively requires good optometric and orthoptic care and includes the use of glasses, contact lenses, eye patching to treat amblyopia, and monitoring of intraocular pressure to detect the development of glaucoma [6,9,11].

Ethical approval for this report was obtained from the Research and Ethics Committee of Lily Hospitals Limited with the approval number LH/HREC-EE-0023-20. Informed consent was also obtained from the patient following the tenets of the Declaration of Helsinki for studies involving human subjects.

## Case presentation

An 18-year-old woman, who was a medical student, presented with a squint and a white spec in the left eye from birth; it was associated with a poor vision for which she had been using spectacles, but with no improvement in her symptoms. The right eye was normal. The patient was a product of normal gestation and full-term delivery.

**Figure 1:**
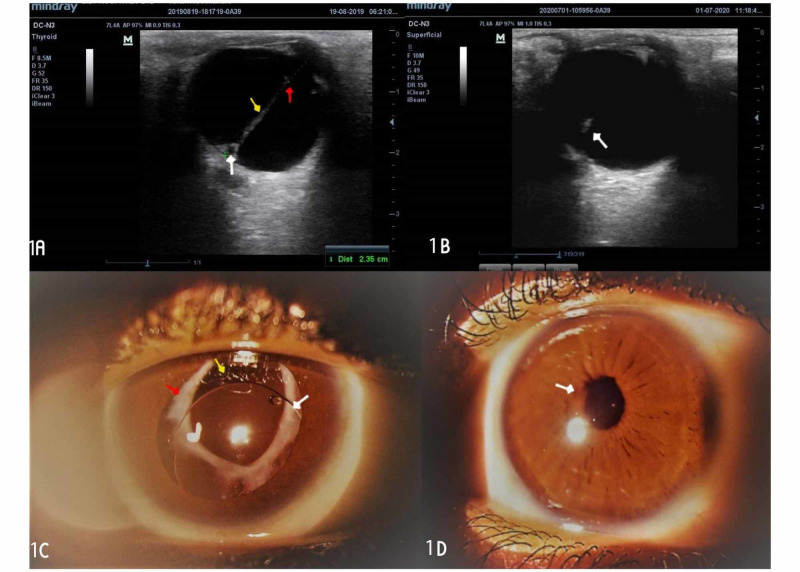
Ultrasound scan of the eye during preoperative and postoperative periods; pupil of the eye after dilatation and during the postoperative period 1A: the image shows the ultrasound scan of the persistent fetal vasculature of mixed morphology (anterior and posterior indicated by a yellow arrow). The posterior part is attached to the retina (Bergmeister’s papillae), indicated by a white arrow, and the anterior part is attached to the posterior capsule of the lens, forming a posterior capsular cataract (Mittendorf dot), indicated by a red arrow. 1B: the image shows a stalk of the lesion postoperatively, indicated by a white arrow. 1C: the image shows an anterior segment photograph of the eye after dilatation with a PMMA intraocular lens implanted into the sulcus during surgery at the one-year follow-up. The white, yellow, and red arrows indicate the sulcus-fixated rigid PMMA intraocular lens, the posterior capsulectomy site exposing the anterior vitreous, and calcified anterior and posterior capsules, respectively. 1D: the image shows the eye before dilatation, at one-year follow-up. The white arrow indicates a round pupil measuring about 2mm in diameter PMMA: polymethyl methacrylate

**Figure 2:**
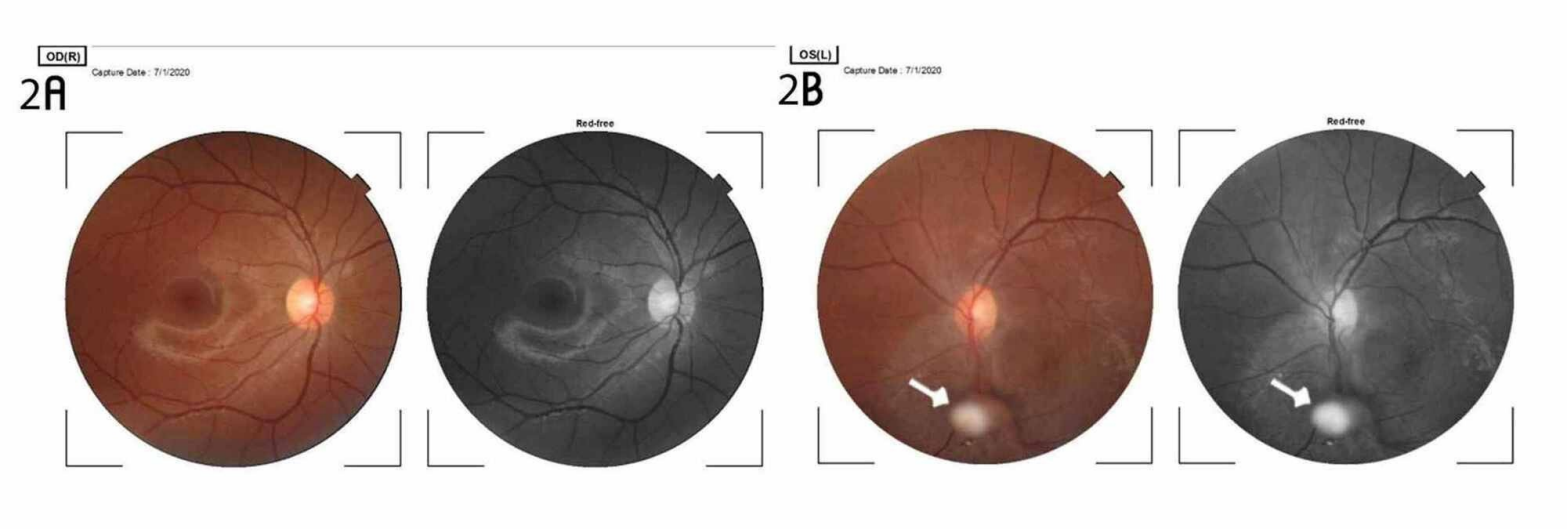
Colored and red-free fundus photographs of both eyes 2A: the image shows the normal findings in the right retina. 2B: the image shows a stump of the excised lesion attached to the inferonasal aspect of the retina in the left eye. The stalk is surrounded by a greyish area that extends superiorly to the optic disc

The patient underwent the following examinations: visual activity, slit-lamp biomicroscopy, intraocular pressure (Goldmann), ophthalmoscopy, fundus photography (OCT TOPCON, 3D OCT-1 Maestro, Topcon, Tokyo, Japan) and Doppler ultrasonography. Intraocular lens (IOL) power was calculated using the Sanders-Retzlaff-Kraff (SRK) II formula. Snellen’s unaided distance visual acuity was 6/4 in the right eye and 6/60 in the left eye. There was no improvement with refraction. Hirschberg's test revealed a 3mm deviation of corneal reflex in the affected eye, which corresponds to an exotropia of 45 prism diopters. Anterior segments were normal; the lens in the left eye had a posterior capsular cataract measuring about 3mm in diameter. Examination of the anterior chamber angles with a four-mirror gonioscopy lens showed open angles up to the ciliary body (Shaffer grade-4) in all quadrants bilaterally. The ultrasound investigation revealed a thick hyperechoic linear band measuring 15.6mm in length from the retina to the posterior lens capsule that showed no flow in color. Doppler interrogation was also performed (Figure 1A). Fundus photography revealed normal findings in the right eye while the left eye revealed a PFV (Figures 2A, 2B). Biometry showed the following values: keratometry K1-43.50D, K2-44.00D, an axial length of 23.6mm, and IOL power of 20.00D. She was worked up for small incision cataract surgery, excision of PFV, anterior vitrectomy, and insertion of a rigid polymethyl methacrylate (PMMA) IOL into the sulcus. She was counseled on the prognosis after surgery and written consent was obtained.

The surgical procedure involved the following steps: cleaning of the periocular skin with 10% povidone-iodine, posterior sub-tenons anesthesia with xylocaine, instillation of 5% povidone-iodine around the forniceal conjunctiva, retraction of the lids with a flexible lid speculum, conjunctival peritomy on the superior aspect of the sclera, and wet field cauterization of bleeding conjunctival vessels. A chevron incision measuring 5mm at the base and 2mm from the corneal limbus was created with a crescent blade and the anterior chamber entered with a keratome knife. The depth of the anterior chamber was maintained with an ocular viscoelastic device when necessary. The anterior capsule of the lens was stained with trypan blue and a continuous circular capsulorrhexis was performed using a cystitome. The inner lip of the scleral tunnel was extended with the keratome knife to 7mm. Hydrodissection was performed with a hydrodissection cannula and the viscoelastic delivery of the nucleus was done. Cortical lens matter was aspirated using Simcoe irrigation-aspiration cannula and the anterior chamber depth was maintained. Posterior capsulotomy was performed around the Mittendorf dot and PFV was excised with intraocular scissors within the vitreous cavity, and anterior vitrectomy was performed through the scleral tunnel. A rigid PMMA posterior chamber IOL (PCIOL) was implanted into the sulcus (Figure 1C). Subconjunctival injection of dexamethasone and intracameral injection of moxifloxacin eye drops were done. The eye was cleaned with gauze and padded till the following day. Postoperative results were satisfactory with a day-one Snellen's visual acuity of 3/60, which improved to 6/60 one week post-op. Postoperative steroid and antibiotic eye drops were administered. The intraocular pressure was normal throughout the follow-up period. Over a year of the follow-up period, the visual acuity remained stable at 6/60 and 6/36, unaided and pinhole, respectively, and the intermittent exotropia resolved from 45 to 30 prism diopters. There was a relief of retinal traction and a resolution of leukocoria. Amblyopia therapy did not improve visual acuity for distance and near. The findings on the ultrasound scan of the eye during the preoperative and postoperative periods are shown in Figures 1A, 1B, respectively. Figure 1C shows the pupil of the eye after dilatation, while figure 1D shows the pupil during the postoperative period with the IOL within the visual axis. Figure 2A shows the fundus photo of the right eye, while Figure 2B shows that of the left eye in the postoperative period.

## Discussion

PFV is an uncommon condition that may be seen in pediatric ophthalmic practice while screening for retinopathy of prematurity and other ocular conditions associated with prematurity. In the newborn, the classical presentation of PFV includes micro-ophthalmia, cataract, and leukocoria [9,13]. PFV is usually unilateral but bilateral when associated with syndromes such as Norrie disease [4,10]. In this case, the patient presented with normal-sized eyes, leukocoria, and a squint at 18 years of age, which suggested that the fundus examination of the eyes had not been performed at birth. PFV does not have any predilection for a particular sex or race or any other risk factors apart from prematurity. The mode of presentation in this patient also suggested a less severe form of PFV. In evaluating a patient with PFV, there is a need for conducting imaging studies in order to rule out a posterior polar cataract. Ultrasonography with Doppler interrogation is indicated to rule out the presence of a viable vessel in the PFV, which could cause a vitreous hemorrhage following surgical intervention. Here, the ultrasound scan showed no blood flow in the PFV. The mechanism of amblyopia in this patient was by stimulus deprivation as the PFV traversed the visual axis.

During the preoperative period, there is a need to discuss the expected outcome of the surgical procedure with the patient. Intraoperatively, care should be taken to ensure minimal distortion of the vitreous humor. In this case, it was achieved by performing a capsulorrhexis on the posterior capsule around the Mittendorf dot and excising the lesion in the vitreous cavity using a pair of Vannas intraocular scissors. After minimal anterior vitrectomy, a 20.00D rigid IOL was implanted into the sulcus [14]. The postoperative care in infants is challenging because of inflammation and amblyopia. Amblyopia results from high anisometropia secondary to unilateral aphakia when an IOL is not implanted at the time of surgery. To achieve a good outcome, there is a need for prompt optometric and orthoptic care as per the recommendations of the amblyopia treatment trials [15]. In this report, we did not expect a significant improvement in visual acuity because of amblyopia that had set in during childhood.

Postoperatively, the fundus photograph showed a stalk of the resected PFV attached to the inferonasal aspect of the retina as seen in Figure 2B. Postoperative outcomes included the resolution of leukocoria, relief of retinal traction, and partial resolution of the squint. The posterior pole was visualized postoperatively and the optic disc and macula showed normal findings. When the visual acuity after surgical intervention for PFV is better than 1/60, it can be referred to as “useful vision”; when it is 1/60 or less, it is considered to be “poor vision” [7]. For the index case, the unaided and aided visual acuity over the follow-up period remained 6/60 and 6/36, respectively, despite amblyopia therapy by patching of the better eye. There was a reduction of the intermittent exotropia from 45 to 30 prism diopters, and the patient’s parents observed that the patient’s eye appeared straighter and no longer deviated like before the surgery. This suggested improved binocular single vision in the patient. This report shows that manual small incision cataract surgery with intravitreal resection of PFV combined with anterior vitrectomy and IOL implantation is a viable option for the management of PFV. The outcome was comparable to that of phacoemulsification and pars plana vitrectomy with the implantation of IOL by competent hands [13].

## Conclusions

The surgical management of PFV is challenging because the visual outcome can be compromised by the type of PFV and the time of intervention. If a case is diagnosed in early childhood, the interventions would yield a better outcome as amblyopia treatment can be initiated. In this case, the diagnosis was made in adulthood at a stage when the eye had developed cataract, amblyopia, and intermittent exotropia, which had caused a cosmetic embarrassment to the patient. In order to achieve an optimal visual outcome, early intervention is therefore advocated for PFV.

## References

[1] Taniguchi H, Kitaoka T, Gong H, Amemiya T (1999). Apoptosis of the hyaloid artery in the rat eye. Ann Anat.

[2] Bari V, Murad M (2003). Persistent hyperplastic primary vitreous. J Pak Med Assoc.

[3] (2020). Persistent hyperplastic primary vitreous. https://eyewiki.org/Persistent_hyperplastic_primary_vitreous.

[4] Payabvash S, Anderson JS, Nascene DR (2015). Bilateral persistent fetal vasculature due to a mutation in the Norrie disease protein gene. Neuroradiol J.

[5] (2020). Pediatric retina: medical and surgical approaches. https://cc.bingj.com/cache.aspx.

[6] Mitchell CA, Risau W, Drexler HC (1998). Regression of vessels in the tunica vasculosa lentis is initiated by coordinated endothelial apoptosis: a role for vascular endothelial growth factor as a survival factor for endothelium. Dev Dyn.

[7] Wang J, Yan H, Du Z, Zhang J, Wang W, Guo C (2020). Atypical anterior persistent hyperplastic primary vitreous: report of a rare case. BMC Ophthalmol.

[8] Hunt A, Rowe N, Lam A, Martin F (2005). Outcomes in persistent hyperplastic primary vitreous. Br J Ophthalmol.

[9] Yusuf IH, Patel CK, Salmon JF (2015). Unilateral persistent hyperplastic primary vitreous: intensive management approach with excellent outcome beyond visual maturation. BMJ Case Rep.

[10] Li L, Fan DB, Zhao YT, Li Y, Cai FF, Zheng GY (2017). Surgical treatment and visual outcomes of cataract with persistent hyperplastic primary vitreous. Int J Ophthalmol.

[11] Nelson L (2011). Pediatric Ophthalmology (Color Atlas and Synopsis of Clinical Ophthalmology).

[12] Zahavi A, Weinberger D, Snir M, Ron Y (2019). Management of severe persistent fetal vasculature: case series and review of the literature. Int Ophthalmol.

[13] Sisk RA, Berrocal AM, Feuer WJ, Murray TG (2010). Visual and anatomic outcomes with or without surgery in persistent fetal vasculature. Ophthalmology.

[14] Lee SM, Yu YS (2004). Outcome of hyperplastic persistent pupillary membrane. J Pediatr Ophthalmol Strabismus.

[15] Kozeis N, Tsaousis KT, Gidaris D (2012). Surgical treatment of persistent fetal vasculature and visual rehabilitation: one-year followup. Case Rep Med.

